# Radiation-dependent demyelination in normal appearing white matter in glioma patients, determined using quantitative magnetic resonance imaging

**DOI:** 10.1016/j.phro.2023.100451

**Published:** 2023-06-01

**Authors:** Anna Ljusberg, Ida Blystad, Peter Lundberg, Emelie Adolfsson, Anders Tisell

**Affiliations:** a Department of Medical Radiation Physics, and Department of Health, Medicine and Caring Sciences, Linköping University, Linköping, Sweden; b Center for Medical Image Science and Visualization (CMIV), Linköping University, Linköping, Sweden; c Department of Radiology, and Department of Health, Medicine and Caring Sciences, Linköping University, Linköping, Sweden

**Keywords:** White matter, Myelin sheath, Follow-up studies, Glioma, Magnetic resonance imaging, Radiotherapy

## Abstract

**Background and purpose:**

A brain tumour, especially a glioma, is a rare disease; it is challenging to treat and the prognosis is often poor. Routine care includes surgery and concomitant chemoradiotherapy (CRT). Diagnostic work-up and treatment effects are typically evaluated using magnetic resonance imaging (MRI). Quantitative MRI (qMRI), unlike conventional MRI, has the advantage of providing tissue-specific relaxation rates and proton density. The purpose is to detect changes in normal appearing white matter (NAWM) in brain tumour patients after CRT using qMRI.

**Materials & methods:**

NAWM was analysed in 10 patients, in 83 MR examinations performed before and after surgery and after CRT. Relaxation rates R_1_ and R_2_, the proton density (PD) and the concentration of myelin (c_My_) were calculated from the qMRI scans and analysed in correlation to radiation dose and time after treatment.

**Results:**

A significant decrease in c_My_ between pre-treatment imaging and first follow-up and an increase in PD were observed. For low doses (less than 30 Gy) PD and c_My_ returned to baseline (=pre-operative status), while for high doses (>30 Gy) the change increased during the full extent of the follow-up period. No difference could be established for R_1_. For R_2_ an increase was observed during the first year, which then gradually returned to baseline. For R_2_, stronger effects were seen as a consequence of higher absorbed doses.

**Conclusion:**

In the long-term follow-up for glioma patients, qMRI is a powerful tool for detecting small changes, such as a decrease of myelin concentration, in NAWM after CRT.

## Highlights

• Changes in white matter after CRT can be detected using relaxometry.
• Changes in white matter are correlated with radiation dose and time after treatment.
• Myelin concentration and proton density in white matter are affected by CRT.

## Graphical abstract

**Figure:**
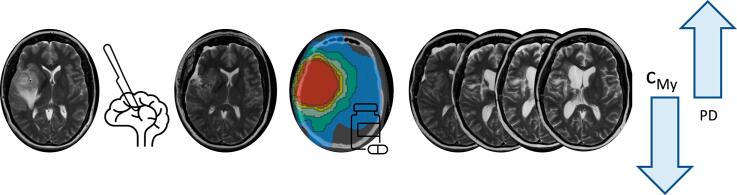

## 1 Introduction

The most common types of brain tumours are gliomas, originating from the glial cells in the brain. Depending on tumour type and malignancy grade, the prognosis varies, with a short overall survival for high-grade gliomas despite an aggressive treatment. After surgery concomitant chemo radiotherapy (CRT) is given with the disadvantage that it affects the surrounding brain tissue as well as the targeted tumour. Also, effects after surgery such as swelling and oedema can cause neurological symptoms and cognitive dysfunctions and these affects are therefore likely to have a long-term impact on quality of life. Damage to the white matter in the brain is often observed within the first year, while radiation effects in the grey matter often do not occur until after six to 12 months [1]. Damage to the grey matter often involves vascular lesions such as telangiectasia and focal haemorrhages, while white matter damage is often transient demyelination [1]. The demyelination is a consequence of the irradiated oligodendrocytes going into apoptosis [2].

For brain tumours and therapy-related affects, MRI is the main imaging technique in clinical practice and includes several images such as T_1_W- and T_2_W-images, T_2_FLAIR, diffusion and perfusion. Assessment of radiological findings is performed according to the RANO criteria ('Response Assessment in Neuro-Oncology Criteria for Gliomas') [3]. To reveal changes and further understand the complicated process in white matter and myelin, diffusion weighted and diffusion tensor imaging has been suggested [4], [5], [6], [7]. Another recent study suggested T_2_ relaxometry for observing changes after proton radiotherapy [8]. Raschke *et* al examined both these techniques for observing changes in normal appearing white matter (NAWM) [6].

To improve imaging of brain tumours further, quantitative MRI (qMRI) has been proposed, as it provides more detailed and quantitative information about tumour properties. One implementation of qMRI is the QRAPMASTER-sequence together with the SyMRI software [9], [10], [11]. This sequence is composed by a combination of interleaved slice selective saturation pulses, and multi echo readouts to generate imaging volumes with a range of echo- and saturation delay- times. These can be used to calculate the qMRI maps; R_1_, R_2_ and PD (R_1_ = 1/T_1_, R_2_ = 1/T_2_). The qMRI maps can be used for creating synthetic images [12], [13], [14], [15]. It can also be used to determine myelin concentrations [16], a method which have been validated using Luxol fast blue staining [17], [18]. A major advantage of the technique is the gain of quantitative and tissue specific values, and also the possibility to perform calculations and statistical tests on these. A disadvantage is the variation of quantitative values depending on age and gender [19], [20]. QMRI maps are also particularly useful for detecting contrast enhancement in peritumoural oedema [21], [22]. Recent published work also concluded that qMRI can be useful for head and neck cancers [23].

The main aim of this project was to investigate if longitudinal qMRI imaging during follow-up after concomitant CRT can be used to detect changes in NAWM. A second aim was to explore if such changes can be related to absorbed dose given in radiotherapy (RT), and if such changes vary over time. The term qMRI will in the following text refer to quantitative values extracted with the QRAPMASTER sequence and SyMRI software.

## 2 Material and methods

### 2.1 Subjects

A retrospective analysis of data collected in the period 2013–2018 was performed on a patient cohort consisting of 25 patients with suspected glioma. The characteristics of the peritumoural tissue specifically has been reported previously [21], [22]. The data collection was approved by the local ethics committee (Dnr: 2011/406-31) and informed consent was obtained. MR scans were performed before and after surgery and as a follow-up after RT completion every third month. Causes for exclusion were: (i) another diagnosis at histopathology (3), (ii) higher dose per fraction (3.4 Gy × 10) (1), (iii) no follow-up MRI after treatment (4) or (iv) not being treated with RT at our institution (7). After exclusion, ten patients remained for final analysis. The characteristics of the ten analysed patients are shown in Table 1. The histopathological diagnoses were classified according to WHO 2007, which was the standard during the time for inclusion [24].

**Table 1:** Characteristics of evaluated patients.

Pat.	Age	Gender	Histopathological diagnosis (WHO 2007)	Dose	Technique	# of follow-up exams	
1	63	M	Anaplastic Oligodendroglioma grade III	2 Gy × 30	VMAT	4	
2	58	F	Glioblastoma	2 Gy × 30	VMAT	4	
3	57	F	Glioblastoma	2 Gy × 30	3DCRT	4	
4	61	F	Glioblastoma	2 Gy × 30	VMAT	8	
5	65	F	Anaplastic Oligodendroglioma grade III	2 Gy × 30	VMAT	9	
6	50	M	Anaplastic Oligodendroglioma grade III	2 Gy × 30	3DCRT	8	
7	68	M	Glioblastoma	2 Gy × 30	3DCRT	3	
8	43	M	Oligodendroglioma grade II	1,8 Gy × 28	3DCRT	7	
9	65	M	Gliosarcoma	2 Gy × 30	VMAT	2	
10	45	F	Glioblastoma	2 Gy × 30	VMAT	8	

### 2.2 Radiotherapy

Nine patients were prescribed the Stupp regime with 60 Gy/30 fractions [25]. One patient, diagnosed with Oligodendroglioma grade II, was treated with 50.4 Gy/28 fractions. The planning target volume, PTV, consists, with margins of 2–3 cm to the cavity from surgery and any residual tumour corrected for anatomical boundaries. Treatment plans were optimized for a mean dose to PTV of 100%, D_98%_ ≥ 95% and D_2%_ ≤ 107% although due to proximity of critical organs such as the brainstem not all plans fulfilled the aims. Treatment was delivered using either a TrueBeam or Clinac IX (Varian, Paulo Alto, San Francisco, USA). For volumetric modulated arc therapy (VMAT) 6 MV was used while for 3D conformal plans (3DCRT) a mixture of 6 MV and 15 MV was used, optimized for every patient. Treatment plans were calculated with the AAA algorithm (vers. 10–13) in the treatment planning system Eclipse (vers. 10–13, Varian, Paulo Alto, San Francisco, USA). Dose delivery was verified with phantom QC measurements for VMAT-plans and by measurements with in-vivo diodes during the first treatment for 3DCRT. The department participates annually in remote audits of machine calibration by IROC-H [26].

### 2.3 MR-acquisition

**Fig. 1:**
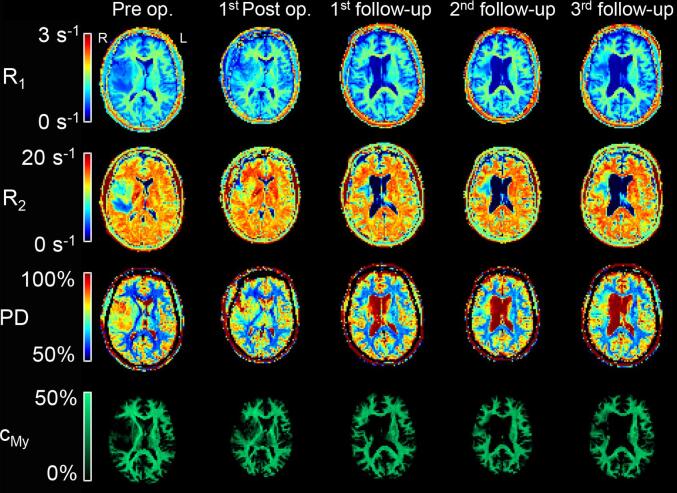
Example of qMRI-maps for one patient. From top to bottom is the qMRI-maps for R_1_, R_2_, PD and c_My_ and from left to right is the images from before and after surgery and the three first follow-up images after RT completion, with approximately three months interval.

MR images were acquired on a 3 T Discovery MR750 (GE Medical Systems, Milwaukee, Wisconsin, US) and qMRI images were processed/calculated using SyMRI (vers 0.45.25/27, SyntheticMR AB, Linköping, Sweden). The QRAPMASTER sequence was added to the clinical protocols for brain tumours. The axial scan has a field of view of 220 × 180 mm^2^ and a voxel size of 0.43 × 0.43 × 5 mm^3^. A total of 24 slices, with 5 mm slice thickness and 1 mm gap, were collected with a scan time of 5:55 min. The qMRI maps (R_1_, R_2_, and PD) and the myelin map (c_My_) were calculated and synthetic T_2_-weighted images reconstructed. In Fig. 1 R_1_, R_2_, PD and c_My_ are shown for one patient from the examinations before and after operation and the first three follow-up examinations after RT completion.

### 2.4 Data analysis

**Fig. 2:**
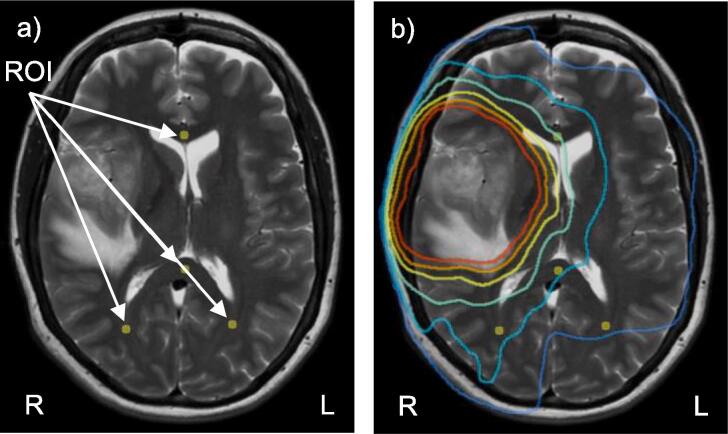
Synthetic T_2_-weighted image with ROI and dose distribution. In a) there is an example of ROI placements in a synthetic T_2_-weighted image of a patient. ROIs are placed in the lower part of the parietal lobe (left and right), in the genu and splenium of the corpus callosum. In b) the same T_2_-weighted image is displayed, but with an overlay of the isodose lines of 10, 20, 30, 40, 50 and 57 Gy.

A total of 83 MRI examinations were concluded for the ten patients during a two-year follow-up period. Out of these, ten were pre-operative examinations (baseline), nine were radicality controls after surgery (1^st^ post op.) and 57 were conducted after CRT (follow-up). On seven occasions extra examinations were done between the radicality control and CRT. These were excluded, leaving 76 examinations for analysis. The number of follow-up examinations varied between two to nine, the follow-up period was up to two years or until death. Circular ROIs with a diameter of 5 mm, 4 mm if the anatomical structure was less than 5 mm (less than 5% of the ROIs), were placed in NAWM in the synthetic T_2_-weighted images by a neuroradiologist (IB). ROI placement was done in the MICE toolkit (vers. 2021.1.0, NONPI Medical AB, Sweden, Umeå). Fig. 2 shows an example of ROIs drawn in one slice. ROIs were placed in up to 14 anatomical regions: the genu and splenium of the corpus callosum, bilaterally in the pedunculus cerebellaris, peritrigonally and in a lower and upper slice of the frontal and parietal lobes. All ROIs were manually placed in a consistent manner to compensate for anatomical shifts in all examinations. However, ROIs were not drawn if the radiologist assessed that an oedema was present in the specific tissue area during the follow-up, or if there was tumour present at baseline. In Appendix A Supplementary data 1, Fig. A.1 displays a histogram of the number of ROIs for each time period and Table A.1 includes the number of ROIs in the different anatomical regions.

The absorbed dose distribution and CT-image were exported from Eclipse and transformed to the qMRI-space by performing a rigid registration of the CT-images to the synthetic T_2_-volume, using the MICE toolkit. For every ROI a mean value of R_1_, R_2_, PD, c_My_ and absorbed dose was extracted, relaxation rates are expressed as per second, PD and c_My_ are expressed in percent and the absorbed dose in Gray. Using Matlab (vers. R2020B, The MathWorks Inc., Natick, MA, USA) the difference in one ROI compared with its baseline value was calculated for all ROIs and time-points. For PD and c_My_ the difference was expressed as the arithmetical difference of percent, percent points (p.p.). Appendix A Supplementary data 2 is an Excel sheet containing all ROIs and their quantitative values and the differences compared with baseline, used for the statistical tests performed.

### 2.5 Statistical tests

Statistical analysis was performed with SPSS Statistics v.28 (IBM Corp, Armonk, USA).

A two-sided paired Student’s *t*-test was used to analyse changes in any quantitative value for all ROIs between baseline and the first follow-up after CRT. Another paired *t*-test evaluated the differences between the pre-operative and the first post-operative images taken for radicality control in order to exclude changes prior to CRT. The paired Student’s *t*-test was used to exclude changes depending on patient, age, gender and anatomical location.

Changes related to absorbed dose and time after concluded CRT were analysed with a mixed model analysis. In this test, each ROI for every patient was seen as a subject in the analysis, with a repeated measurement at approximately every third month. The dependent variable (the difference in R_1_, R_2_, PD or c_My_) were assumed to be linearly dependent on the covariates absorbed dose (Gy) and time (months), both of which were assumed to be fixed effects. Random effects depending on patient and anatomical location were accounted for in the mixed model. Significance level was set to 0.017, to correct for multiple comparison of three different statistical tests.

## 3 Results

Comparisons of the differences in R_1_, R_2_, PD and c_My_ between the baseline image and the first follow-up show a significant difference in all parameters except R_1_. The mean value of difference in R_1_ is – 0.013 s^−1^ (p-value 0.13). For R_2_ the difference is 0.33 s^−1^, for PD it is 1.5 percentage points (pp) and the difference in c_My_ is −2.2 pp, all with a p-value less than 0.001. There was no significant difference between the pre-operative and radicality control images. The mean values of these differences are printed in Table 2.

**Table 2:** Student’s *t*-test of differences from baseline for immediate post-operative examination and 1^st^ follow-up after RT. There was no significant difference between baseline and the first post-operative MRI. Significant changes in PD, c_My_ and R_2_ were observed during the first follow-up after RT completion. The difference is expressed as the arithmetic difference between the two percentages, percent point (p.p.), for PD and c_My_ and in per second for the relaxation rates.

	1^st^ post op. mean of difference *(n = 104)*	p-value	1^st^ follow-up mean of difference *(n = 116)*	p-value
PD (p.p.)	0.32	ns	1.5	<0.001
c_My_(p.p.)	−0.55	ns	−2.2	<0.001
R_1_ (s^−1^)	−0.008	ns	−0.013	ns
R_2_ (s^−1^)	−0.11	ns	0.33	<0.001

The results of the mixed model test are printed in Table 3. There is a significant correlation with time and absorbed doses for all parameters except R_1_ where only time is significant. For an absorbed dose of 50 Gy, the contribution to the difference of R_2_ corresponded to 0.35 s^−1^, PD 1.3 p.p. and c_My_ decreased by 2.2 p.p. The effect of time in the model corresponded to a difference in R_1_ of 0.024 s^−1^, R_2_ −0.20 s^−1^, PD −0.50 p.p. and c_My_ 0.80 p.p at 12 month.

**Table 3:** The covariate factors and the significance of the mixed model test, the time factor is per month and the dose factor per Gray. PD and c_My_ are expressed as 10^−3^ and the arithmetic difference between the two percentages, percent point (p.p.) and the relaxation rates are expressed as 10^−3^ per second.

	Time factor (month^−1^)	p-value	Dose factor (Gy^−1^)	p-value
PD (10^−3^p.p.)	−42	0.005	26	<0.001
c_My_ (10^−3^p.p.)	67	0.002	−42	<0.001
R_1_ (10^−3^s^−1^)	2	<0.001	0	ns
R_2_ (10^−3^s^−1^)	−17	<0.001	7	<0.001

**Fig. 3:**
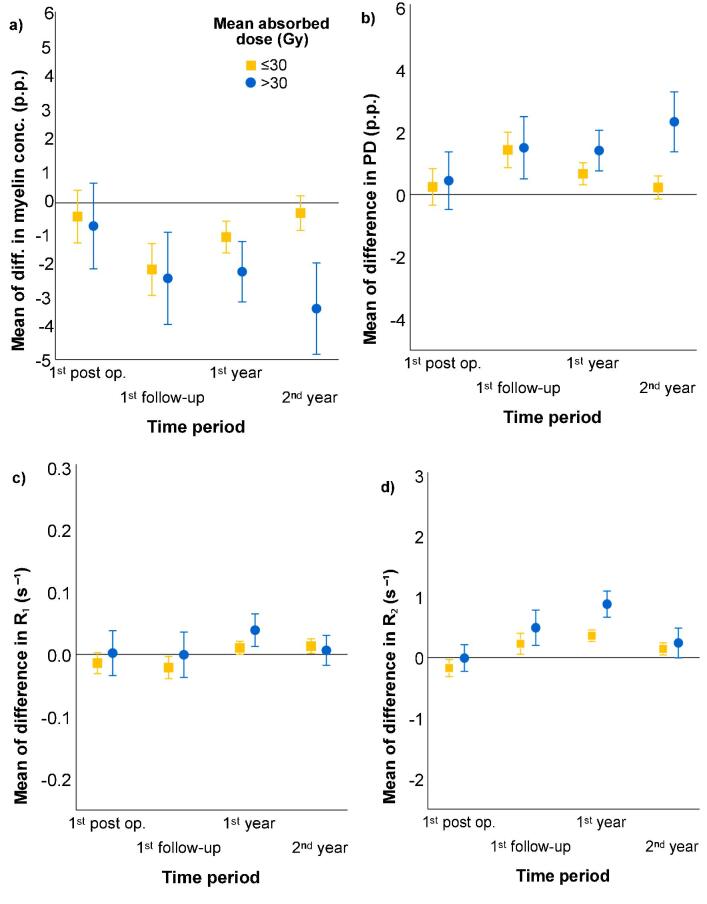
Mean difference for different time period and absorbed dose. Mean differences for doses less (yellow square) or more (blue circle) than 30 Gy for different examination times. In panel a) the difference in percent points in myelin concentration is displayed, b) contains the difference of percent points in proton density, and c) and d) are the difference per second of relaxation rates R_1 _and R_2_. The error bars indicate the 95% confidence interval for mean value of ROIs differences. (i) A decrease of 2% was observed for c_My_, during the first follow-up. This was then returned to baseline for low doses, but not for higher doses during the follow-up period. (ii) An increase in PD was observed during the first follow-up, followed by a subsequent return to baseline for low doses. (iii) In R_1_ there were very small differences for all doses regardless of time-point. (iv) In R_2_ an increase was detected, with a maximum difference during the first year, before returning to baseline. For higher doses a larger increase in R_2_ was observed.

A visual presentation of the mean values of change from baseline and the 95% confidence interval for mean value of ROIs difference for all quantitative parameters is found in Fig. 3. In the plot there is a distinction between ROIs receiving low or high doses with a threshold of 30 Gy.

## 4 Discussion

The aim of this work was to conclude if qMRI could be useful for detecting changes in NAWM in brain tumour patients after CRT. For this purpose two different statistical test were performed on the quantitative data that had been acquired. The results of the Student's *t*-tests confirm that differences can be detected and that the changes did not occur due to surgery but due to CRT. The result concluded no significant changes during the radicality control although significant differences were found during first follow-up after CRT completion in all parameters except R_1_, see Table 2. The correlation test with time and absorbed dose showed that there was a significant dependence on time after RT in all quantitative values: R_1_, R_2,_ PD and c_My_. In addition, there was also an absorbed dose dependency in all parameters, except in R_1_.

In Fig. 3 the time and dose dependency are shown with mean values of the differences for two different dose levels and time points. For low doses, less than 30 Gy, the difference decreased with time and was restored to baseline, while for higher doses, above 30 Gy, there was no turning point, and the difference increased with time during the follow-up period. R_1_ displayed the smallest differences and appeared to be stable over time, even though with somewhat larger differences for higher doses. R_2_ had the same appearance over time for both low and high doses, but the differences were more pronounced for higher doses. In the baseline examination 117 unique ROIs were drawn. It would be of interest to illustrate the changes over time in narrower dose intervals, unfortunately there is too few ROI for this. The threshold of 30 Gy was chosen based on results in other studies, mainly with DTI, where changes have been observed depending on dose, with narrower bins, and were a higher magnitude or a steeper gradient for doses above 30 Gy was observed [5], [6], [7]. The linear dependency used in the mixed model may not be the best fit, as seen in Fig. 3, since changes could appear and disappear over time and between examinations. This behaviour is hard to predict with a model and more data is needed for validating a more advanced model instead of a linear.

The brain tissue is changed as a consequence of aging and the quantitative parameters are therefore affected [19], [20], [27]. Apart from age also sex and anatomical location in the brain effect the quantitative values as well as different diseases and therapies. Post treatment changes in volume of the whole brain, grey or white matter have been reported previously [28], [29], [30], [31]. There are also publications focusing on the use of diffusion imaging techniques for observing white matter affects, however, reports on changes in relaxation rates after radiotherapy are rare [4], [5], [6], [7]. The myelin sheath consists of lipid and cholesterol rich layers with water in between the layers. This water is referred to as myelin water and has a very short T_2_. With multi compartment analysis of T_2_, changes in concentration of myelin water can be detected. The cholesterol on the other hand affects the T_1,_ with a shortening of T_1_ in myelin rich areas [32], [33]. One example where multicomponent T_2_ relaxometry was used was a study by Bontempi *et* al., in which an increase in myelin water with increased dose after proton radiotherapy was observed [8]. Another example is Raschke *et* al. who in addition to using diffusion tensor imaging also quantified T_1_, T_2_* and PD and found a significant reduction of T_2_* in white matter studying a similar population of patients as in the cohort examined here. The QRAPMASTER-sequence has a too long echo time for performing effective component analysis as alluded to above, however using SyMRI the c_My_ could nevertheless be determined. If, the observed decrease in c_My_ was due to demyelination we would anticipate a more prominent effect of R_1_, although such was not observed. If the change instead was due to an increase in extracellular water concentration, but not identified as oedema by a radiologist, R_2_ would be affected. Independent of the techniques used for observing changes on white matter, it is nevertheless challenging to explain the causality of the changes that were observed. Although a rather small difference was observed in myelin concentration (−2.2% at first follow-up) these changes are at least ten times larger than natural ageing for a person of age 58 (which was the mean age in this study) [20].

This study has some limitations. The changes observed could have been caused by other confounding factors than just dose and time, such as medication and the time point of the MRI examination. Changes in quantitative values could also have occurred between the MRI scans and therefore remained unobserved. The total number of ROIs included in the statistical analysis decreased with time due to progression of the disease and decease of patients. After one year only five patients contributed to the analysis.

In conclusion, this study shows that by using QRAPMASTER and SyMRI we could detect dose dependent changes in white matter. This illustrated the value of qMRI for evaluating changes in brain tissue after RT, however, from the data available it was hard to conclude the biological cause of the observed changes. In future studies a larger group of patients could be included. Additionally, an improved qMRI pulse sequence moving from 2D- to a 3D-acquisition would be beneficial as it would increase spatial resolution by decreasing the slice thickness from 5 mm to 1.2 mm [34]. This would decrease the influence of partial volume effects, and would thereby also allow for a quantification of smaller anatomical structures.

## Declaration of Competing Interest

The authors declare that they have no known competing financial interests or personal relationships that could have appeared to influence the work reported in this paper.
