# Supplementary material for: Radiation-dependent demyelination in normal appearing white matter in glioma patients, determined using quantitative magnetic resonance imaging
Source: Phys Imaging Radiat Oncol. 2023 Jun 1;27:100451. doi: 10.1016/j.phro.2023.100451 (PMC10500023; doi:10.1016/j.phro.2023.100451)
Supplement: Supplementary data 1 [file mmc1.pdf]

Table A.1: Location and number of ROI drawn in normal appearing white matter during the follow-up period (immediate post-operative examination for radicality control not included). A total of 662 ROIs.

ROI location	Total number of ROI	
	Left	Right
Pedunculus cerebellaris	57	57
Peritrigonal	41	20
Lower frontal lobe	46	50
Lower parietal lobe	50	35
Upper frontal lobe	46	50
Upper parietal lobe	49	48
Genu	51	
Splenium	62	

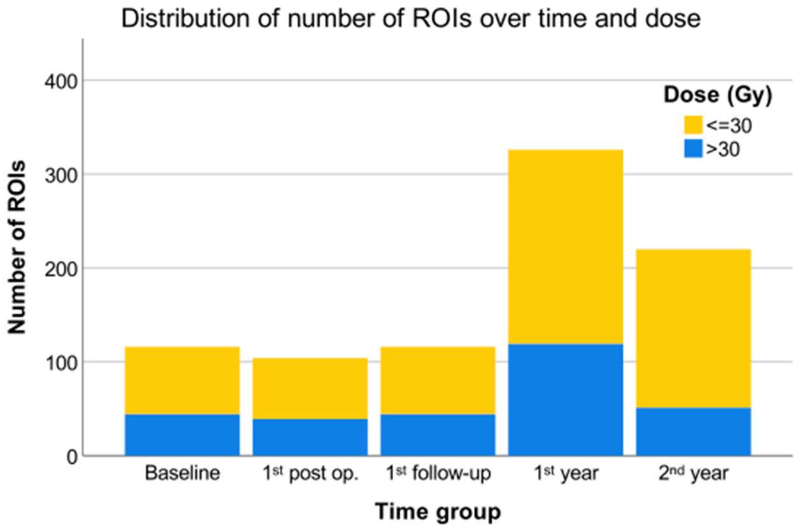

Figure A.1: Histogram of mean absorbed dose to ROI in different times after therapy.
